# Improvement in Sensitivity of an Inductive Oil Palm Fruit Sensor

**DOI:** 10.3390/s140202431

**Published:** 2014-02-03

**Authors:** Norhisam Misron, Noor Hasmiza Harun, Yeoh Kian Lee, Roslina Mohd Sidek, Ishak Aris, Hiroyuki Wakiwaka, Kunihisa Tashiro

**Affiliations:** 1 Faculty of Engineering, University Putra Malaysia, 43400 Serdang, Selangor, Malaysia; E-Mails: noorhasmiza@bmi.unikl.edu.my (N.H.H.); yeoh.kian.lee@gmail.com (Y.K.L.); roslina@eng.upm.edu.my (R.M.S.); ishak@eng.upm.edu.my (I.A.); 2 Institute of Advanced Technology (ITMA), Universiti Putra Malaysia, 43400 Serdang, Selangor, Malaysia; 3 Faculty of Engineering, Shinshu University, Wakasato 4-17-1, Nagano, 380-8553, Japan; E-Mails: wakiwak@shinshu-u.ac.jp (H.W.); tashiro@shinshu-u.ac.jp (K.T.)

**Keywords:** inductive, resonant frequency, air coil, oil palm, frequency characteristics, maturity classification, inductive concepts

## Abstract

Among palm oil millers, the ripeness of oil palm Fresh Fruit Bunch (FFB) is determined through visual inspection. To increase the productivity of the millers, many researchers have proposed with a new detection method to replace the conventional one. The sensitivity of such a sensor plays a crucial role in determining the effectiveness of the method. In our preliminary study a novel oil palm fruit sensor to detect the maturity of oil palm fruit bunches is proposed. The design of the proposed air coil sensor based on an inductive sensor is further investigated to improve its sensitivity. This paper investigates the results pertaining to the effects of the air coil structure of an oil palm fruit sensor, taking consideration of the used copper wire diameter ranging from 0.10 mm to 0.18 mm with 60 turns. The flat-type shape of air coil was used on twenty samples of fruitlets from two categories, namely ripe and unripe. Samples are tested with frequencies ranging from 20 Hz to 120 MHz. The sensitivity of the sensor between air to fruitlet samples increases as the coil diameter increases. As for the sensitivity differences between ripe and unripe samples, the 5 mm air coil length with the 0.12 mm coil diameter provides the highest percentage difference between samples and it is amongst the highest deviation value between samples. The result from this study is important to improve the sensitivity of the inductive oil palm fruit sensor mainly with regards to the design of the air coil structure. The efficiency of the sensor to determine the maturity of the oil palm FFB and the ripening process of the fruitlet could further be enhanced.

## 1. Introduction

Generally, the quality of oil palm fruits is categorized based on the texture, shape and color of the fruit [1]. Currently in Malaysia, the human expert grading approach is used to inspect the maturity of oil palm FFB and classify them for harvesting. Typically, the color of the surface of the fruit and the number of loose fruit drops from bunches are the two main factors that guide the judgement of human experts [1,2]. In practice, this type of grading method often leads to mistakes where there is high potential to grade the fruit wrongly. Besides that, different humans have different judgements, which means that grading results from different human experts will differ. Furthermore, human inspection is a time-consuming method. These factors often lead to considerable profit losses [3,4]. Therefore, an automated fruit grading system is highly required. An automated fruit grading system must be rapid, accurate and reliable. Apart from that, any oil palm grading system should not destroy the palm oil FFB during the analysis [5,6].

In the past few years, a number of different automated fruit grading systems were proposed and tested. The most popular is the use of the color vision system that requires an advanced digital camera to capture pictures of oil palm FFBs and a computer for analysis [7–10]. An artificial intelligence system is sometimes used together with the color vision system to classify the oil palm FFBs [11–15]. Overall, this method requires complicated algorithms and precise image collection for the recognition stages and it produced an average success rate of 73.3% [11,16–18].

Another method used by researchers is the assessment using the RGB space. This oil palm grading method uses spectral analysis based on the different wavelengths of red, green and blue color of the image [19,20]. In this method, the color quality of the image is relatively important. Classifications of the ripe category within the bunch for the average value of red component were successfully carried out. However, the red components for unripe and under ripe categories cannot be differentiated and the average success rate for this method is reported as 49% [21]. Other disadvantages of this method are that this type of classification has to be performed indoors [22,23].

Analyzing the moisture content of oil palm fruits is another grading method. The moisture content of the mesocarp in the fruit affects the surface color and the weight of the oil palm fruit. Microwave moisture sensors are used to investigate the moisture content of oil palm fruits [24–28]. However, the measurement procedure is quite complicated and time-consuming.

Aside from these, Magnetic Resonance Imaging (MRI) and bulk Nuclear Magnetic Resonance (NMR) are other methods used by researchers to monitor the development and ripeness of oil palm FFBs [29]. FFB samples are harvested at different Week After Anthesis (WAA). Then, both equipments are used to measure the continuous change in spin-spin relaxation times of protons of the water and lipids for the development of a ripening tracking process for FFBs. The changes between oil and moisture content in the oil palm FFBs are observed based on the differences in their spin-spin relaxation times and significant results are obtained. This method requires the usage of complicated and expensive equipment. In addition to that, skilled personnel are needed to operate it, limiting the testing to be done indoors.

Another type of imaging method involves Non-destructive near Infra Red (NIR) spectroscopy. Two NIR spectrometers are used to scan the oil palm fruits with different modes. After that, chemical contents of palm oil are analyzed by using Partial Least Squares Regression (PLSR) models [30].

Besides imaging technology, a capacitive-based concept of grading method is proposed in [31]. The capacitive concept is applied to measure the dielectric properties of the oil palm fruit. This measurement method yielded 5% accuracy for the dielectric constant (ɛ′) and 3% for the dielectric loss (ɛ″). The capacitive method is similar to the other methods, where supporting equipment is needed and it is not suitable for outdoor testing.

In the prevailing research works, no grading methods using an inductive concept is proposed. An inductive concept non-destructive grading method is proposed in this research work, based on the moisture content of the oil palm fruit. The permeability value of water is 1.2566270 × 10^−6^. Thus, with a low permeability value compared to other materials, such as metals, a high frequency range is used in the measurement. This proposed inductive method has great potential for use in outdoor testing [32–34]

In this paper, investigation on the oil palm ripeness sensor based on the resonant frequency (*f*_r_) is presented. Inductance values in the high frequency range are used to determine the ripeness of the oil palm fruits which are then categorized into ripe and unripe fruits. The frequency characteristics of the sensors are studied and the *f*_r_ of air (*f*_ra_), ripe fruit (*f*_rr_) and unripe fruit (*f*_ru_) are analyzed. Initially, the value of *f*_rr_ and *f*_ru_ is normalized to *f*_ra_. Then, the deviation between the mean value in the normalized resonant frequency (N*f*_r_) between the air (N*f*_ra_) and ripe fruit (N*f*_rr_) as well as between air and unripe fruit (N*f*_ru_) are observed and analyzed for the effect of the size of the sensor and the coil diameter size affecting the sensitivity of the sensor, which is determined by the deviation in the mean value between N*f*_ra_ and N*f*_rr_ as well as in between N*f*_ra_ and N*f*_ru_. The larger the deviation from the mean value the more sensitive is the sensor. In this study, twenty sensors with different sensor sizes as well as different coil diameter sizes are built. Looking into the effects of coil diameter, the results portray a uniform pattern throughout the testing. It is observed that the N*f*_rr_ leads the N*f*_ru_. The value of the N*f*_r_ decreases as the air coil length is increased. As for the effects of air coil length, the differences between the ripe to unripe samples increase as the air coil length increases. The results from this study play an important role in designing the air coil structure as it will improve the sensitivity of the oil palm sensor to determine the maturity of the oil palm FFB as well as the ripening process of the fruitlets. Nevertheless, the inductive oil palm ripeness sensor method offers a few advantages such as it is a passive type sensor, reduces time consumption and is an accurate grading system. With the advantages provided by this oil palm ripeness sensor, it is believed that the inductive method would be a good alternative method to grade oil palm fruits. The disadvantages of the sensor will be avoided with a better insulated design in future work. The result from the study should be useful for future research in designing an oil palm ripeness sensor based on the inductive concept.

## 2. Basic Principles

### 2.1. Structure

**Figure 1.**
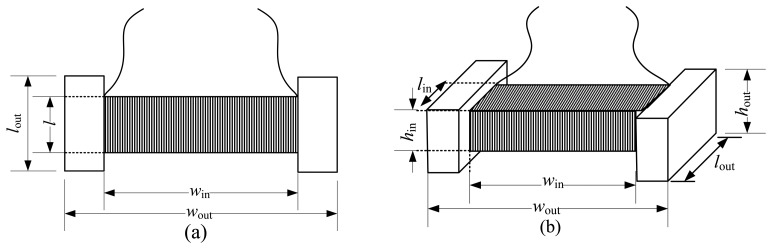
Air coil flat-type shape structure (**a**) top view (**b**) 3D view.

**Figure 2.**
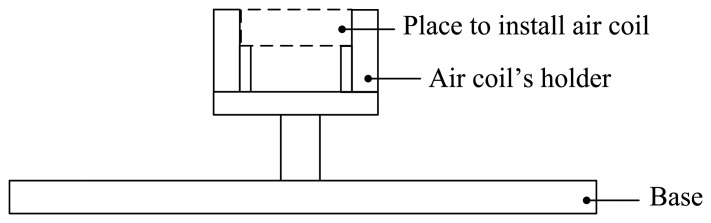
Oil palm ripeness sensor holder.

The oil palm ripeness sensor is shown in Figure 1. The oil palm fruit sensor has a rectangular shape. The main parameters of the air coil are its height (outer height, *h*_out_ and inner height, *h*_in_) and width (outer width, *w*_out_ and inner width, *w*_in_), which remain constant throughout the experiment. As for length, the outer length, *l*_out_ and the inner length, *l* are varied by 1 mm for each type of sensor. Therefore, there are four types of sensor with different lengths being used in the experiment. All four sensors are designated by their inner air coil lengths, *l* which are 2, 3, 4 and 5 mm, being tested to observe the effects of the air coil length besides the effects of the coil diameter. To hold the sensor tight and without displacement, the sensor is placed in a holder. Figure 2 shows the holder, where the space with dotted lines is the place where the sensor is to be placed. The fabrication of the sensor uses the plastic Perspect, a non-conducting material that minimizes the flux disturbance in the sensor.

Assuming these four sensors represent a set of sensor, a total of five sets sensors are built and wound with copper wire coil diameter of 0.10, 0.12, 0.14, 0.16 and 0.18 mm. Therefore, there a total of 20 sensors were used in these investigations. Table 1 shows all of the sensors that are designed. Since the sensors are very small in size, it is not possible to wind the coil with a machine. Therefore, all of the sensors are wound by hand. To minimize the error when measurements are taken, the winding of the copper coil is kept as close as possible, reducing the gaps between windings.

**Figure 3.**
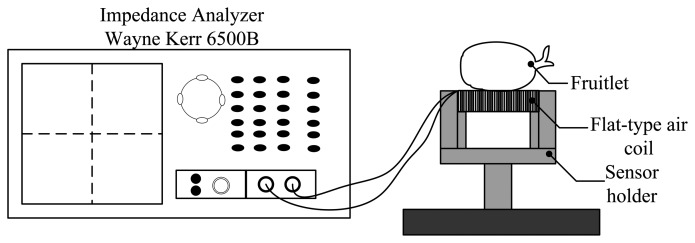
Experimental setup for the flat-type air coil.

The investigation started with the arrangement shown in Figure 3. Twenty samples of fruitlets from two categories; ripe and unripe, are tested between 20 Hz to 120 MHz. The Wayne Kerr 6500B impedance analyzer is used to measure the inductance, the resistance and the resonant frequency of each air coil. There are five different sizes of copper wire diameter used in this study ranging from 0.10 to 0.18 mm. The number of turns of the air coil is fixed at 60 turns. The impedance analyzer with the setup as in Table 2 has been standardized throughout the measurement period as well as for all samples.

### 2.2. Samples

**Figure 4.**
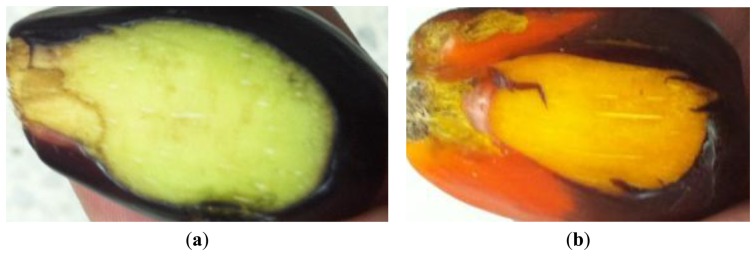
Fruitlet samples (**a**) unripe (**b**) ripe.

**Figure 5.**
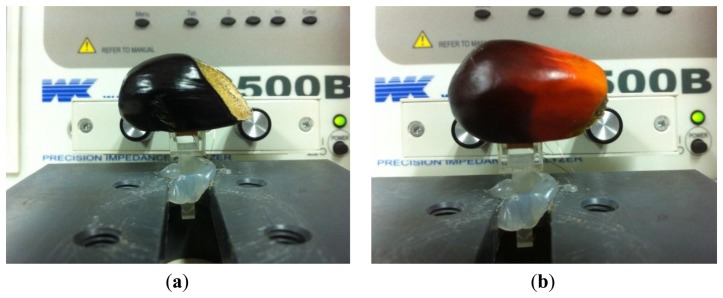
Measurement set-up of the resonant frequency of fruit samples (**a**) unripe (**b**) ripe.

Twenty samples from two categories of oil palm fruitlets (unripe fruitlets and ripe fruitlets) were tested in this investigation. Each fruitlet represents each sample. Therefore, twenty samples consisting of ten ripe fruitlets and ten unripe fruitlets were used in the experiment. Each selected category is based on the surface color of the fruitlets. The unripe fruitlets are dark purple in color while the ripe fruitlets are orange in color, as shown in Figure 4. For the unripe fruitlets, the samples are selected during the 7th week after anthesis (WAA) as in Figure 4a and for the ripe fruitlets on the 15th week after anthesis (WAA) as in Figure 4b [33]. On top of that, the size of the fruitlets is chosen based on the diameter of the air coil for the sensor. Each sample is selected and plucked from the same oil palm Fresh Fruit Bunch (FFB) on the day of testing. This is to ensure that the sample used is fresh and free from any physical contamination. The gap between the fruit and the sensor is minimized in order to get precise results. Therefore, the fruit is cut as in Figure 5 so that the surface contact with the sensor is flat and no gap exists between the fruit and the sensor.

### 2.3. Normalization of Resonant Frequency

The sensitivity of the sensors looks into two aspects; firstly the sensitivity diffwerence between air and samples and the sensitivity difference between ripe to unripe fruitlets. This is done initially by normalizing the resonant frequency of all sensors for samples to the resonant frequency of air. The sensitivity difference between air and fruitlets is related to the differences in the mean value of the N*f*_r_ air to the samples. The sensitivity difference between samples is defined as the deviation in the mean value of the N*f*_rr_ and N*f*_ru_ as in Equation (1):

(1) $$\text{Sensitivity between samples}\;=\;\text{N}f_{\text{r>r}}-\;\text{N}f_{\text{ru}}$$

The sensor is highly sensitive when the deviation of the normalized resonant frequency between ripe and unripe fruit is huge. The mean value for each normalized resonant frequency is calculated for air and fruitlet samples before the deviation between both been determined. The deviation between both air and fruitlet samples can be illustrated by two methods. Firstly, either through direct interpretation or with a percentage difference value. Therefore, it is important to find out the differences of resonant frequency between air and ripe fruits and also air and unripe fruits. To do this, the normalized value of the resonant frequency of air, ripe fruits and unripe fruits are calculated by the equation shown below:

To calculate the normalized frequency for air:

(2) $$\text{Normalized frequency for air}=\frac{\text{Resonant frequency for air}}{\text{Resonant frequency for air}}$$

This indicated that normalized frequency for air is always equal to 1.

To calculate the normalized frequency for ripe fruit:

(3) $$\text{Normalized frequency of ripe fruit}=\frac{\text{Resonant frequency of ripe fruit}}{\text{Resonant frequency of air}}$$

To calculate the normalized frequency for unripe fruit:

(4) $$\text{Normalized frequency of unripe fruit}=\frac{\text{Resonant frequency of unripe fruit}}{\text{Resonant frequency of air}}$$

Equations (2)–(4) are used to calculate the normalized frequency for air, ripe fruit and unripe fruit. By conducting the calculation and finding out the normalized frequency for air, ripe fruit and unripe fruit, the differences in mean value between the air and ripe fruit as well as between air and unripe fruit can be obtained. Thus, the sensitivity of the each sensor can be determined.

## 3. Results and Discussions

### 3.1. Resonance Characteristics

**Figure 6.**
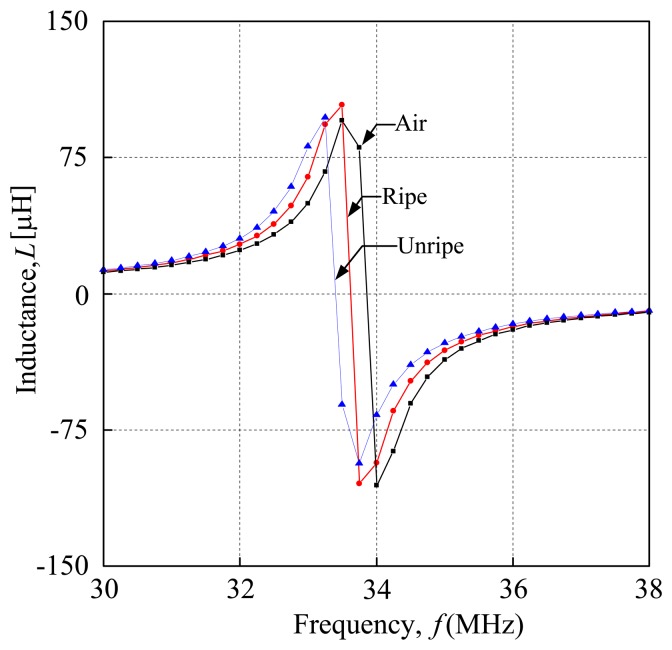
Inductance characteristics of the oil palm fruit sensor.

When analyzing the inductance characteristics of air, ripe fruits and unripe fruits, the graph gives different resonant frequencies, where the resonant frequency refers to the peak of the frequency. Although air, ripe fruit and unripe fruit are giving different resonant frequencies, the graphs show similar curves. Figure 6 shows the general result when running the measurement procedures within a range of frequency. The x-axis represents the frequency and the y-axis represents the inductance value. From the graph, it is obvious that a different frequency point, gives an inductance value. At the resonant frequency, the inductance value is the highest. The shape of the graph is shown in Figure 6, where the black line is the characteristic inductance of air, the red line is the characteristic inductance of ripe fruit while the blue line is the characteristic inductance of unripe fruit. The inductance characteristics of the each sensor portray a similar pattern throughout the whole series of experiments. It is important to standardize the inductive characteristics of each sensor to ensure the repeatability of the sensor. In this paper, the value of the resonant frequency of each sample and air is recorded and plotted against the air coil length and the coil diameter. The value of the resonance is then normalized agianst the resonance frequency of air. It is important to determine the sensitivity of the sensor based on the normalized resonance frequency for air and the fruitlet samples.

### 3.2. Normalization of the Resonance Frequency

**Figure 7.**
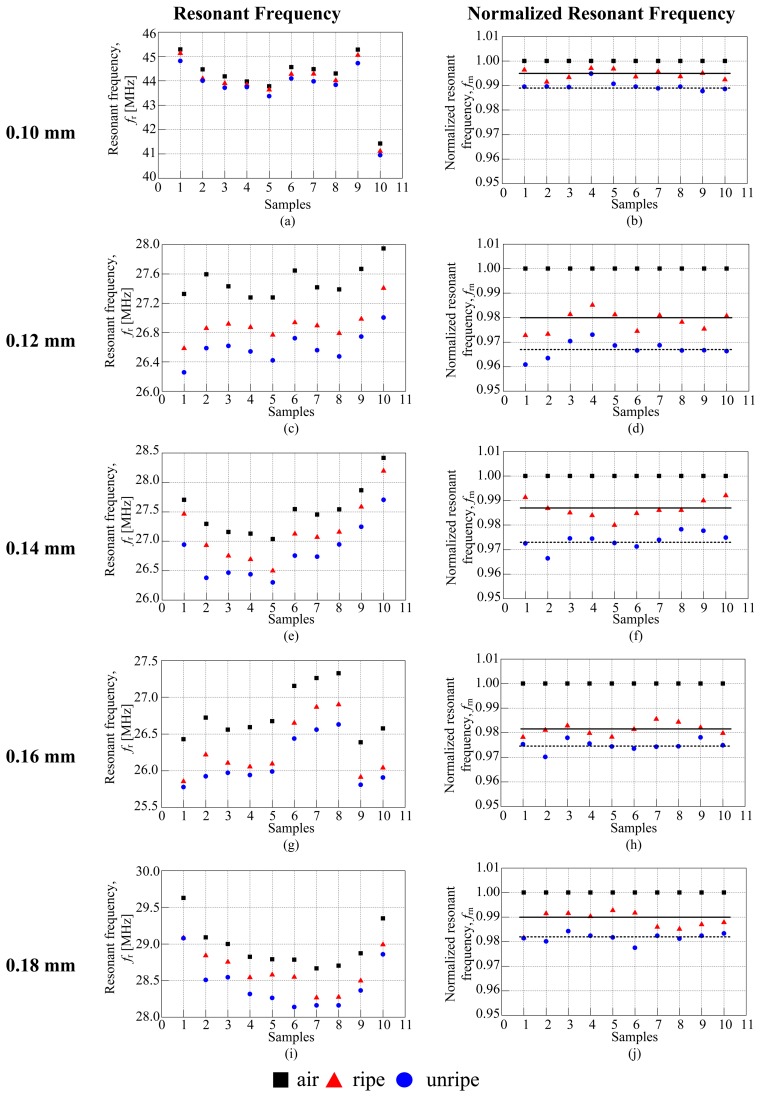
The normalized resonant frequency for 5 mm air coil length.

The experiments conducted for all twenty sensors of various air coil lengths and coil diameters are targeted to collect the resonance characteristics of the sensor. The resonance frequency is then plotted against the air coil length and coil diameter to investigate the effects of both on the resonance characteristics of the sensor. The value of the resonance frequency of fruitlet samples is then normalized to the value of the resonance frequency of air. Among all twenty sensors, the 5 mm air coil length for various coil diameters are selected to be highlighted in Figure 7. The 5 mm air coil length shows promising and outstanding results compared to the other air coil lengths used in this investigation. The resonant frequencies for all ten samples for each category are plotted. Figures 7a,c,e and g show the resonance frequencies for all the samples. The graph shows a similar pattern for all samples, regardless of the coil diameter. The resonance frequency of air leads the resonance frequency of ripe and unripe samples. To clarify the observation, the resonance frequency for each type of coil diameter is then being normalized to the resonance frequency of air. Figures 7b,d,f and h show the graph of the normalized resonance frequency. The value of the normalized resonance frequency is then further analyzed with the calculation of the mean value between ripe to unripe samples. The mean value for each coil diameter is presented as dotted lines on the normalized resonance frequency graph. From Table 3, it can be observed that the 5 mm air coil length with the 0.14 mm coil diameter shows the highest mean value which is 12.9 × 10^−3^. It is then followed by the 0.12 mm coil diameter sensor whose mean value is 11.2 × 10^−3^. Results from the normalized resonant frequency is then further discussed and analyzed in determining the sensitivity of the sensor to ripe and unripe samples. In this research, the sensitivity is defined as the deviation between the mean values of ripe to unripe samples.

### 3.3. Effects of Coil Diameter

**Figure 8.**
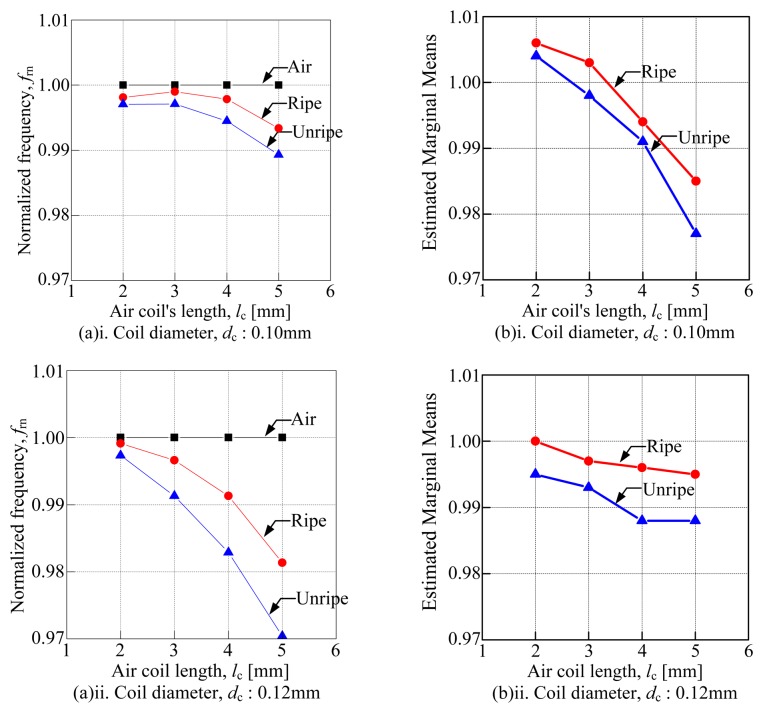

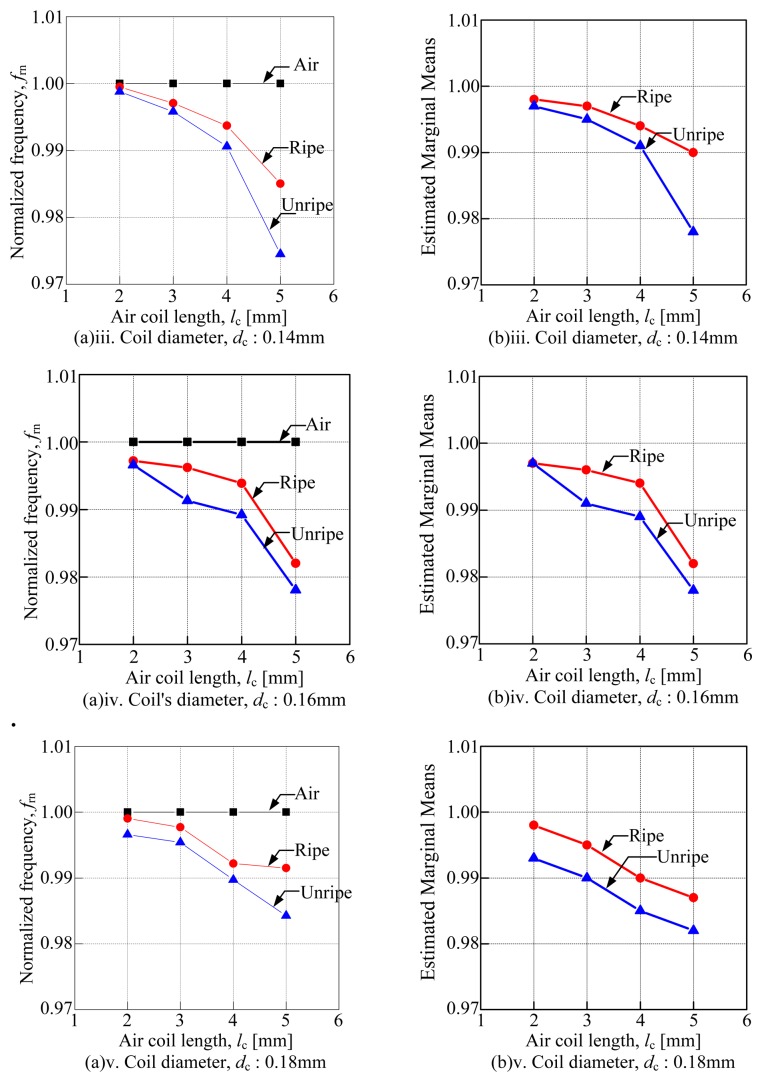
Effects of coil diameter. (**a**) The N*f*_r_ for coil diameter *versus* air coil length, (**b**) The estimated marginal means *versus* air coil length.

Figure 8 shows the effects of coil diameter ranging from 0.10 to 0.18 mm. It was looking into the N*f*_r_ versus the air coil length calculated manually as shown in Figure 8a and the estimated marginal means versus air coil length as shown in Figure 8b. The estimated marginal means was plotted based on the results obtained using two way ANOVA. The effects of coil diameter portray a uniform pattern throughout the air coil length as seen in Figure 8 for both methods. The N*f*_r_ of ripe samples dominates the N*f*_r_ for unripe samples. Generally, the value of the N*f*_r_ seems to decrease as the air coil length is increased. Table 4 shows the deviation between ripe to unripe samples which has been calculated manually for a 5 mm air coil length while Table 5 shows the standard deviation for each type of sensor calculated using ANOVA. The deviation is calculated in order to analyze the sensitivity of each sensor, looking into the effects of the coil diameter. Table 4 shows that the 5 mm air coil length with 0.12 mm coil diameter yields the highest deviation between ripe to unripe samples. Therefore, the result reflects the sensitivity of the 5 mm air coil length with 0.12 mm coil diameter.

### 3.4. Effects of Air Coil Length

**Figure 9.**
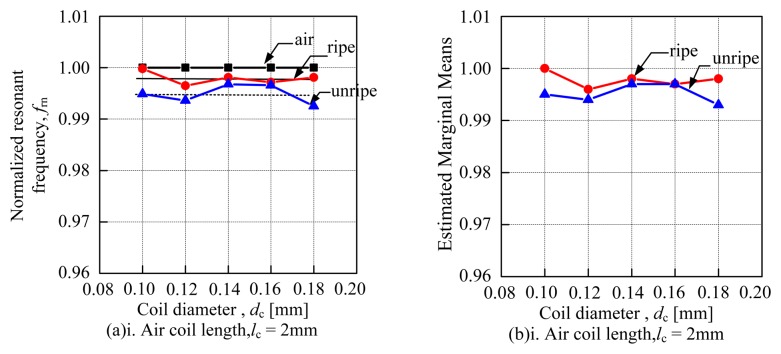

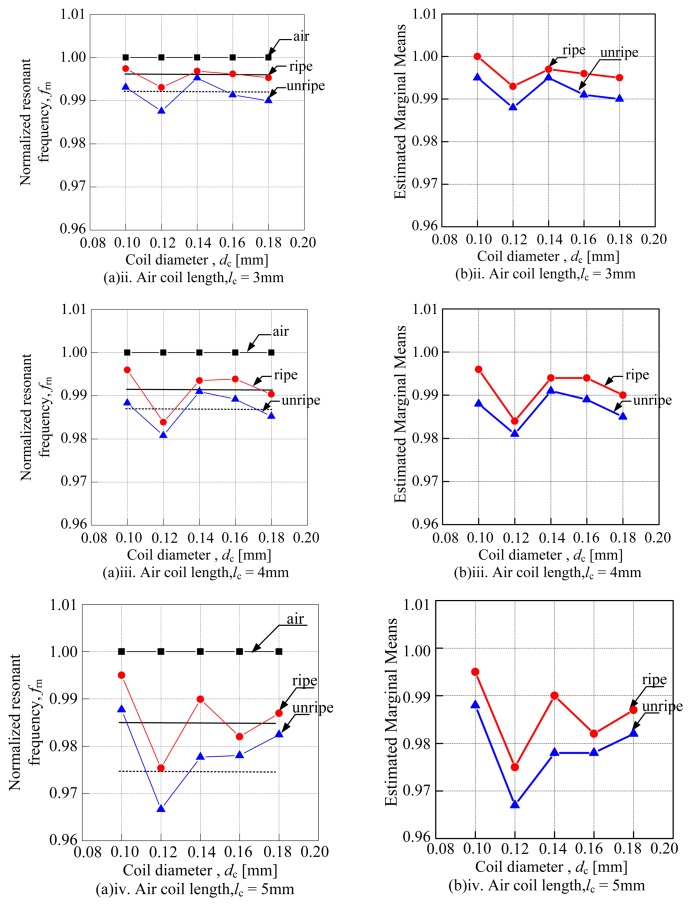
Effects of air coil length. (**a**) The N*f*_r_ for air coil length *versus* coil diameter, (**b**) The estimated marginal means *versus* coil diameter.

Figure 9 shows the effects of air coil length ranging from 2 to 5 mm. It was looking into the N*f*_r_ *versus* the coil diameter calculated manually as shown in Figure 9a and the estimated marginal means *versus* coil diameter as shown in Figure 9b. The mean value of the ripe samples and unripe samples are calculated and illustrated by the dotted line on the graph. Then, the deviation between both mean values is calculated and tabulated in Table 6. In Table 7, the standard deviation for each type of sensor which has been calculated using the two way ANOVA was displayed. Both methods are conducted to evaluate the sensitivity of each sensor by looking into the effects of the air coil length. From Table 6, it can be seen that the deviation between the ripe and unripe samples increases as the air coil length increases. The 5 mm air coil length yields the highest deviation amongst the mean values of the samples. Therefore, generally it can be concluded that the sensitivity of the sensor increases as the air coil length increases.

### 3.5. Sensitivity Analysis

**Figure 10.**
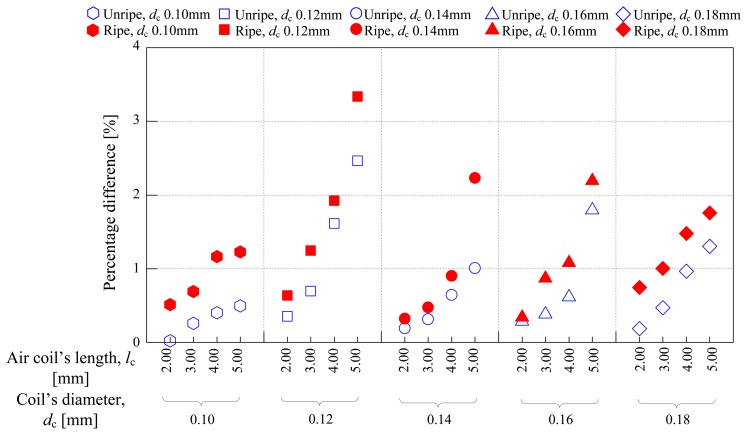
Percentage difference of various coil diameter and air coil length.

This section has the most important role in this study whereby the results from Sections 3.3 and 3.4 were combined and analyzed on the same graph. It is important to observe both effects—coil diameter and air coil length—in order to improve the sensitivity of the air coil. As explained in Section 2.1, the air coil plays the important role for the inductive oil palm sensor. Therefore, the effects of coil diameter and air coil length were observed to improve the sensitivity of the air coil. Figure 10 shows the overall characteristics of the twenty types of sensors used in this investigation. The percentage difference calculated manually between the air and fruitlet samples is plotted *versus* air coil length, *l*_c_ and coil diameter, *d*_c_. The characteristic of the graph covers two different parameters, namely the air coil length, *l*_c_ and the coil diameter, *d*_c_. Firstly, looking into the sensitivity of the sensor between air and fruitlet samples, unripe and ripe, from the graph, it can be clearly seen that as the air coil length increases, the percentage difference between air and fruitlet samples increases. However, for 5 mm air coil length sensor, when the coil diameter varies from 0.18 mm to 0.10 mm, the optimum value shoots up at the coil diameter of 0.12 mm. Looking into sensitivity between ripe and unripe fruitlet samples, the mean values of percentage difference for ripe and unripe samples have been calculated. Then, the deviation between both mean values for coil diameter of 0.10 mm to 0.18 mm are calculated and shown in Table 8. From Table 8, the sensor with a coil diameter of 0.10 mm shows the highest deviation value, followed by 0.18 mm and 0.12 mm coil diameter. Regardless of the highest deviation value shown by the 0.10 mm coil diameter, it is observed that the 0.12 mm coil diameter shows a convincing slope of percentage difference as shown in Figure 10 for all types of air coil length. The 0.12 mm coil diameter provides the highest percentage difference between sample and it is amongst the highest deviation values between samples as tabulated in Table 6. Therefore, based on the analysis from Sections 3.3 and 3.4, it can be concluded that the 5 mm air coil length sensor with the coil diameter of 0.12 mm could be chosen as the most sensitive air coil in this study. Other than that, the estimated marginal means calculated using the two ways using ANOVA was performed in this section as in Sections 3.3 and 3.4. However, the graph plotted from the result obtained from ANOVA is exactly the same as shown in Figure 10. Therefore, the graph in Figure 10 was used to represent the overall characteristics of the air coil for both methods; manual and ANOVA.

## 4. Conclusions

This paper presents an investigation on the effects of air coil structure, mainly the air coil length and coil diameter, on the sensitivity of the oil palm fruit sensor. The sensitivity of the oil palm fruit sensor is looking into two aspects, the sensitivity differences between air and fruitlet samples and the sensitivity differences between ripe and unripe fruitlets. Looking into the effects of coil diameter, the result portrays a uniform pattern throughout the testing. The normalized resonant frequency of ripe samples, N*f*_rr_ dominates the normalized resonant frequency for unripe samples, N*f*_ru_. The value of the normalized resonant frequency, N*f*_r_ decreases as the air coil length increases. As for the effects of air coil length, the mean value of the percentage difference between ripe to unripe fruitlet samples is evaluated and the deviation between both has been calculated. The difference between the ripe to unripe samples increases as the air coil length increases. As for the conclusions, the sensitivity of the oil palm fruit sensor has been determined. The sensitivity of the sensor between air to fruitlet samples increases as the coil diameter decreases, except for 0.10 mm coil diameter. This could be due the skin effects that occur between windings. For the sensitivity differences between ripe and unripe samples, the 5 mm air coil length with the 0.12 mm coil diameter provides the highest percentage difference between samples and it is amongst the highest deviation values between samples. Therefore, the 5 mm air coil length with the 0.12 mm coil diameter can be concluded as the most sensitive air coil in this study. It is important to further improve the sensitivity of the air coil in order to enhance the efficiency of the inductive oil palm sensor to determine the maturity of the oil palm FFB, especially for the fruitlet ripening process. Thus, this study directs improvements to the design of the air coil sensor to enhance the potential of the inductive coil resonant frequency technique for determining the maturity of oil palm fruitlets, as well as the oil palm fresh fruit bunch to maximize the production of palm oil.

## Figures and Tables

**Table 1.** Detailed dimensions for the air coil.

**Item**	**Dimension [mm]**			
Inner length, *l*	2	3	4	5
Outer length, *l*_out_	3	4	5	6
Outer width, *w*_out_	8	8	8	8
Inner width, *w*_in_	6	6	6	6
Outer height, *h*_out_	2	2	2	2
Inner height, *h*_in_	1	1	1	1

**Table 2.** Specification for frequency characteristics experimental setup.

**Parameter/Part**	**Value/Type**
Type of measurement setup	Series (*L*_s_–*R*_s_)
Voltage (V)	0.5
Frequency (MHz)	20–120
Sweep (points)	800
No of turns	60
Coil diameter (mm)	0.10–0.18

**Table 3.** The deviation between mean value of ripe samples and unripe samples for 5 mm air coil length.

**Coil Diameter, *d*_c_ [mm]**	**Mean Value of Ripe Samples**	**Mean Value of Unripe Samples**	**The Deviation Between Ripe to Unripe (× 10^−3^)**
0.10	0.994	0.989	4.73
0.12	0.978	0.967	11.2
0.14	0.986	0.973	12.9
0.16	0.981	0.974	6.47
0.18	0.988	0.981	6.84

**Table 4.** The deviation between ripe to unripe samples for 5 mm air coil length.

Coil diameter, *d*_c_ [mm]	0.10	0.12	0.14	0.16	0.18
The deviation between ripe to unripe samples	0.405	1.094	1.053	0.624	0.724

**Table 5.** The standard deviation value for all types of sensors calculated using ANOVA.

**Coil Diameter,** ***d*_c_** **[mm]**	**Air Coil Length,** ***l*_c_** **[mm]**	**Standard Deviation**
0.10	2	0.00202
	3	0.00387
	4	0.00220
	5	0.00616

0.12	2	0.00354
	3	0.00283
	4	0.00566
	5	0.00495

0.14	2	0.00091
	3	0.00112
	4	0.00182
	5	0.00865

0.16	2	0.00042
	3	0.00346
	4	0.00331
	5	0.00281

0.18	2	0.00395
	3	0.00378
	4	0.00363
	5	0.00321

**Table 6.** The deviation between the mean values of ripe samples and unripe samples.

Air coil's length, *l* [mm]	2	3	4	5
The deviation between ripe to unripe samples (× 10^−3^)	3.05	4.32	4.62	7.36

**Table 7.** The standard deviation value for all types of sensors calculated using ANOVA.

**Coil Diameter,** ***d*_c_** **[mm]**	**Air Coil Length,** ***l*_c_** **[mm]**	**Standard Deviation**
2.00	0.10	0.00354
	0.12	0.00141
	0.14	0.00071
	0.16	0.00000
	0.18	0.00354

3.00	0.10	0.00354
	0.12	0.00354
	0.14	0.00141
	0.16	0.00354
	0.18	0.00354

4.00	0.10	0.00540
	0.12	0.00220
	0.14	0.00182
	0.16	0.00331
	0.18	0.00363

5.00	0.10	0.00517
	0.12	0.00616
	0.14	0.00865
	0.16	0.00281
	0.18	0.00321

**Table 8.** The mean value of the percentage difference between ripe to unripe samples.

Coil diameter, *d*_c_ [mm]	0.10	0.12	0.14	0.16	0.18
The deviation between ripe of unripe sample	0.604	0.503	0.441	0.353	0.515
